# A modular theory of multisensory integration for motor control

**DOI:** 10.3389/fncom.2014.00001

**Published:** 2014-01-31

**Authors:** Michele Tagliabue, Joseph McIntyre

**Affiliations:** Centre d'Étude de la Sensorimotricité, (CNRS UMR 8194), Institut des Neurosciences et de la Cognition, Université Paris Descartes, Sorbonne Paris CitéParis, France

**Keywords:** sensory integration, motor control, maximum likelihood, reference frames

## Abstract

To control targeted movements, such as reaching to grasp an object or hammering a nail, the brain can use divers sources of sensory information, such as vision and proprioception. Although a variety of studies have shown that sensory signals are optimally combined according to principles of maximum likelihood, increasing evidence indicates that the CNS does not compute a single, optimal estimation of the target's position to be compared with a single optimal estimation of the hand. Rather, it employs a more modular approach in which the overall behavior is built by computing multiple concurrent comparisons carried out simultaneously in a number of different reference frames. The results of these individual comparisons are then optimally combined in order to drive the hand. In this article we examine at a computational level two formulations of concurrent models for sensory integration and compare this to the more conventional model of converging multi-sensory signals. Through a review of published studies, both our own and those performed by others, we produce evidence favoring the concurrent formulations. We then examine in detail the effects of additive signal noise as information flows through the sensorimotor system. By taking into account the noise added by sensorimotor transformations, one can explain why the CNS may shift its reliance on one sensory modality toward a greater reliance on another and investigate under what conditions those sensory transformations occur. Careful consideration of how transformed signals will co-vary with the original source also provides insight into how the CNS chooses one sensory modality over another. These concepts can be used to explain why the CNS might, for instance, create a visual representation of a task that is otherwise limited to the kinesthetic domain (e.g., pointing with one hand to a finger on the other) and why the CNS might choose to recode sensory information in an external reference frame.

## 1. Introduction

Reaching to grasp an object requires that the CNS compare the position and orientation of the object with the position and orientation of the hand in order to generate a motor command that will bring the hand to the object. Depending on the situation, the CNS might use more than one sensory modality, such as vision and proprioception, to sense the position and orientation of the target and of the hand, with each source of information encoded in its own intrinsic reference frame. This raises the question as to how the CNS combines these different sources of information to generate the appropriate motor commands.

**Figure 1:**
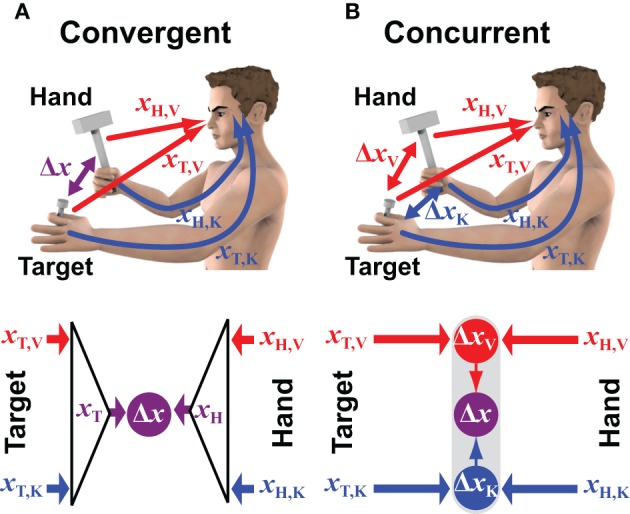
**Convergent vs. concurrent models of sensorimotor integration**. The two conceptual models are applied to a case in which visual (V) and kinesthetic (K) information can be used to estimate the positions of both the target (T) and the hand (H) that hold the hammer. Red and blue colors correspond to information encoded in retinal and body centered reference frames, respectively. **(A)** In the convergent model the visual and kinesthetic information about the target (*x*_T,V_ and *x*_T,K_, respectively) are optimally combined to build a multimodal estimate of its position (*x*_T_). Ditto for the hand/hammer position (*x*_H_). The two optimal estimates are then compared (subtracted) to compute the movement vector Δ*x*. **(B)** In the concurrent approach the positions of the target and the hand/hammer are compared simultaneously in visual and kinesthetic space. The two resulting unimodal movement vectors Δ*x*_V_ and Δ*x*_K_ are then optimally combined to compute the multimodal movement vector Δ*x*.

One school of thought contends that processes of sensor fusion for perception can be explained by the tenets of optimal estimation and control. According to the principles of maximum likelihood estimation, sensory signals that contain redundant information should be combined based on the expected variability of each so as to maximize the probability of producing a value close to the true value of what is being measured. This concept has been used with success in recent years to explain how humans combine different sources of sensory information to generate robust estimates of the position, size and orientation of external objects (Landy et al., 1995; Ernst and Banks, 2002; Kersten et al., 2004; Kording et al., 2007). Of greater interest for us, however, is the task of reaching an object with the hand, which adds additional aspects to the process beyond that of simple perception. The position and orientation of the object and of the hand must be effectively subtracted at some level, be it to compute a movement vector during task planning or to apply corrective actions based on real-time feedback during the course of the movement.This aspect of the task immediately brings to mind two additional issues that must be resolved: (1) To compare the position and orientation of two entities, sensory information about each must be expressed in a common coordinate frame. What reference frame(s) are used to perform the requisite computations? (2) The fusion of redundant sensory information might occur at various stages in the perception-action cycle. Where and how are the principles of maximum likelihood applied? In this article we will contrast two possible models of sensor fusion, which we will call *convergent* and *concurrent*, as illustrated in Figure 1 for the task of hitting a nail with a hammer.

The convergent model shown in Figure 1A reflects the conventional idea that the CNS constructs a single representation of the target based on all available sensory information. In the example of hammering a nail, this includes the position of the nail-head in the visual field and the position of the fingertips holding the nail as sensed by kinesthesia. Weighting can be used to privilege either the visual or the kinesthetic information in the estimate of the target position; ditto for the estimation of the hammer's position and orientation, for which both visual and kinesthetic information are available. The combined representations are then compared in some reference frame that could be the reference frame intrinsic to one of the sensory modalities, or it could be some other, more generalized coordinate system. For instance, kinesthetic information could be transformed into retinal coordinates, or both visual and kinesthetic information could be transformed into a common reference frame centered on the head or on the trunk or referenced to external objects (McIntyre et al., 1997; Guerraz et al., 1998; Henriques et al., 1998; McIntyre et al., 1998; Carrozzo et al., 1999; Pouget et al., 2002a; Avillac et al., 2005; Obhi and Goodale, 2005; Byrne et al., 2010). Under this scheme, the CNS would combine all available sensory information about the target into a single, optimal representation of its position and orientation. Similarly, sensory information would be combined to form an optimal representation of the hand's position and orientation in the same general reference frame. The comparison of target and hand would then be carried out within this general reference frame and the difference between the two positions would be used to drive the motor response.

Figure 1B shows the alternative hypothesis by which the CNS performs a distributed set of concurrent comparisons within each reference frame first, and then combines the results to form a unique movement vector (Tagliabue and McIntyre, 2008, 2011, 2012, 2013; McGuire and Sabes, 2009, 2011; Tagliabue et al., 2013). In the example of hammering the nail, visual information about the nail-head is compared to visual information about the hammer while at the same time kinesthetic information about the hand holding the nail is compared with kinesthetic information about the hand swinging the hammer. Each comparison is carried out separately and thus may be carried out within the coordinate system intrinsic to the corresponding sensory modality. Under this formulation, a movement is programmed based on an optimal combination of the different movement vectors within each of the various reference frames. In this way the CNS accomplishes multimodal sensorimotor coordination in a modular fashion by performing a number of simpler target-hand comparisons in parallel.

The purpose of this article is to examine in greater detail these two hypotheses of convergent versus concurrent comparisons of target and hand for reaching movements, both at a theoretical level and through a targeted review of the pertinent literature. In section 2 we differentiate further the two models at the conceptual level by showing mathematically how the application of optimal estimation differs between them. Using these equations, we go on to present the experimental evidence supporting the hypothesis that the CNS functions according to the concurrent model. In section 3 we examine the conditions in which the CNS will transform information from the intrinsic reference frame of one sensor to the reference frame of another. Key to this discussion is an assessment of how coordinate transformations and memory processes affect the variability of the outcome, and we explicitly take into account how co-variation of transformed signals affects the choice of weighting. Section 4 examines the time course of the underlying sensorimotor processes, providing insight into when sensorimotor transformations are actually performed and, as a corollary, indicating that not only does the CNS perform multiple comparisons in parallel, it maintains parallel memory traces in multiple reference frames as well. In section 5 we generalize the concepts of convergent and concurrent processes to more than two sensory modalities, and in section 6 we use these formulations to consider trade-offs between using sensory information encoded in reference frames intrinsic to the sensors themselves or with respect to extrinsic reference frames such as the visual surrond or with respect to gravity. In the final section we describe some specific predictions made by different concurrent and convergent formulations and discuss how the models might be differentiated experimentally.

## 2. Multiple, concurrent vs. multimodal, convergent

The two models depicted in Figure 1 can be described mathematically, in the linear case, as a set of weighted sums and differences. We use here linear formulations because they simplify the equations and are sufficient to make predictions about how the two models might differ computationally and experimentally. The main feature of the convergent model in Figure 1A is that a single representation of the target is compared to a single representation of the hand in the common reference frame, and a movement is performed that reduces the difference of these two estimates, Δ*x*, to zero. The equation describing this formulation is:

(1) $$\Delta x=\left(w_{\text{T},\text{V}}x_{\text{T},\text{V}}+w_{\text{T},\text{K}}x_{\text{T},\text{K}}\right)-\left(w_{\text{H},\text{V}}x_{\text{H},\text{V}}+w_{\text{H},\text{K}}x_{\text{H},\text{K}}\right)$$

where *x*_T,V_ and *x*_T,K_ represent the position of the target detected by vision and kinesthesia, respectively, *x*_H,V_ and *x*_H,K_ represent the detected position of the hammer in each of those reference frames, and *w*_T,V_, *w*_T,K_, *w*_H,V_ and *w*_H,K_ are the weights given to each of these pieces of information. In the concurrent model of Figure 1B, target and hand are compared in the reference frame of each sensory modality first, and then the final movement vector Δ*x* is computed as a weighted sum of the individual differences. This process can be described by the equation:

(2) $$\Delta x=\lambda_{\text{V}}\text{​}\left(x_{\text{T},\text{V}}-x_{\text{H},\text{V}}\right)+\lambda_{\text{K}}\text{​}\left(x_{\text{T},\text{K}}-x_{\text{H},\text{K}}\right)$$

where λ_V_ and λ_K_ represent the weight given to the comparisons carried out in each of the two sensory modalities. Common to both Equations (1 and 2) is the idea that redundant information from the various sensory modalities can be weighted differently through the factors *w* and λ. In fact, Equation (2) is a special case of Equation (1), with the added constraint that within each sensory modality, signals about the target and the hand must have the same weight:

(3) $$w_{\text{T},i}=w_{\text{H},i}=\lambda_{i}$$

In the linear formulation used here, therefore, the computational difference between the two models is not so much in terms of the order in which sensory information is added or subtracted, but rather in terms of how the weighting factors *w* and λ are chosen.

The principles of maximum likelihood estimation (MLE) can be applied to both Equations (1 and 2) to find weighting factors that are in some sense optimal, although they differ in terms of what is optimized. The optimal estimation of a parameter *p* given noisy measurements (*m*_1_,…,*m*_*n*_) corresponds to the value that maximizes the probability distribution *P*(*m*_1_,…,*m*_*n*_|*p*) which for independent measurements is equal to $P(m_{1},\ldots ,m_{n}|p)=\prod_{i\;=\;1}^{n}P(m_{i}|p)$. If each measurement is considered to be governed by Gaussian noise, the optimal estimate is analytically derived to be the weighted average such that the relative weight given to any one of the component quantities is equal to the inverse of it's variance relative to all the other quantities:

(4) $$w_{m_{i}}=\frac{\sigma_{m_{i}}^{-2}}{\sum_{i\;=\;1}^{n}\sigma_{m_{i}}^{-2}}$$

where σ^2^_*m*_*i*__ is the variance of measurement *m*_*i*_. Thus, noisy variables are given less weight compared to those that are more reliable (Ghahramani et al., 1997). If weighted in this manner, the linear combination of different sources of information results in a reduction of output variability (i.e., an increase in movement precision) compared to the use of any one source of information alone. For illustration purposes, therefore, we assume that the noise exhibited by each sensory signal is Gaussian so that we may apply the linear maximal likelihood solution (Equation 4) to find the optimal weights.

For the convergent model in Figure 1A, applying MLE in order to compute the weighting factors (*w*′s) in Equation (1) means that an optimal estimate of the position of the hand, derived from all available sensory feedback about the hand, will be compared to (subtracted from) an optimal estimate of the target's position, similarly derived from all available sources of sensory information about the target. Applying Equation (4) to the convergent model, the sets of weights for *i* = K and *i* = V are:

(5) $$w_{\text{T},i}=\frac{\sigma_{\text{T},i}^{-2}}{\sigma_{\text{T},\text{V}}^{-2}+\sigma_{\text{T},\text{K}}^{-2}}\text{and }w_{\text{H},i}=\frac{\sigma_{\text{H},i}^{-2}}{\sigma_{\text{H},\text{V}}^{-2}+\sigma_{\text{H},\text{K}}^{-2}}$$

The computation of weighting factors (λ′s) for the parallel structure in Figure 1B is somewhat different. Here, target and hand are compared in both sensory modalities in parallel (Δ*x*_*i*_ = *x*_T,*i*_ − *x*_H,*i*_) and maximum likelihood then determines how much weight should be given to each of these comparisons, based on the expected variance of each of the computed differences. Given that the variance of a difference is simply the sum of the variances of its minuend and of its subtrahend (σ^2^_Δ_*i*__ = σ^2^_T,*i*_ + σ^2^_H,*i*_) and applying Equation (4), the weight given to each difference is computed as:

(6) $$\lambda_{i}=\frac{\sigma_{\Delta_{i}}^{-2}}{\sigma_{\Delta_{\text{V}}}^{-2}+\sigma_{\Delta_{\text{K}}}^{-2}}$$

Conceptually, therefore, the convergent and concurrent models differ primarily in terms of what is optimized. For the convergent model, an optimal estimate of the target and an optimal estimate of the hand are computed and then used to compute a movement vector. Under the concurrent model, multiple movement vectors are computed and then these vectors combined in an optimal fashion. Thus, even though Equations (1 and 2) are algebraically very similar, the choice of what to optimize when determining the various weights leads different results for the two different models. Note that the neural system may not operate in a strictly linear fashion, in which case differentiating between the two model structures would be even more important in terms of model predictions. But even the linear analysis presented here allows one to draw a distinction between the convergent and concurrent models, both conceptually, as we have described here, and experimentally, as we will show in the following paragraphs.

### 2.1. Distinguishing between models

When both target and hand can be localized via all the same sensory modalities, the convergent and modular formulations differ very little in terms of the predicted outcomes. In the example of hitting a nail with a hammer, this corresponds to the situation in which one can simultaneously see and feel with the hand both the hammer and the nail. In these circumstances, both models predict that more weight will be given to the most reliable (e.g., the least noisy) sensory channels. However, when only a subset of sensory information is available (e.g., only vision of the target or only kinesthesia about the hand), the two different formulations predict two substantially different outcomes.

**Figure 2:**
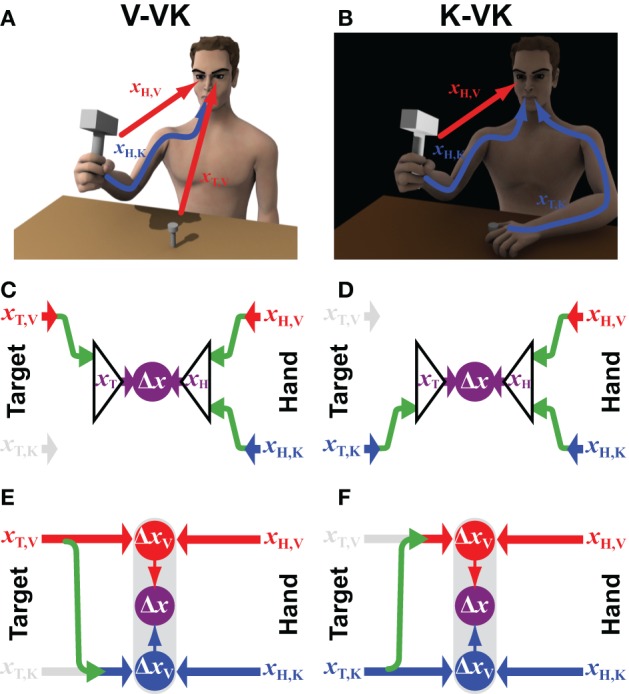
**Incomplete sensory information**. The computational structure of the convergent and concurrent models are compared for situations in which the target position can be sensed through **(A)** visual (*x*_T,V_) or **(B)** kinesthetic (*x*_T,K_) information only, whilst information from both sensory modalities (*x*_H,V_ and *x*_H,K_) can be use to estimate the effector/hand position. Panels **(C,D)** represent how available sensory signals would be used following the Convergent Model in each of the two situations, respectively. Panels **(E,F)** illustrate the computational structure of the Concurrent Model for the same two situations. Green arrows represent the cross-modal sensory transformations that might be performed. Grayed out symbols indicate sensory inputs that are absent, as compared to the situation shown in Figure 1. All other notations and color conventions are the same as in Figure 1.

Consider the situation of a nail that is already imbedded in the wall, such that it need not be held by the non-dominant hand (Figure 2A). Information about the target would therefore be limited to the visual domain. Compare this to hammering a nail that is held by the non-dominant hand, but whose head is obscured from view (Figure 2B). This example is perhaps not a very wise thing to do in real life, but it illustrates the point. To generalize, we will refer to these two types of tasks by the notation V-VK (visual target, visual and kinesthetic hand feedback) and K-VK, respectively, and to the original case of hammering a hand-held nail with full vision of both target and hands as a VK-VK task. In the case of the convergent model (Figures 2C,D), the lack of one source of information about the target simply means that an optimal combination of the remaining sensory cues will be used to localize the target. Thus, in V-VK, a representation of the target based on visual cues, transformed into the common reference frame, will be compared with a representation of the hand in that same reference frame derived from both visual and kinesthetic feedback. Similarly, in K-VK a representation of the target derived from kinesthetic information will be compared with a representation of the hand that is based on an optimal combination of visual and kinesthetic cues.

Applying the concurrent scheme to the situations shown in Figures 2A,B, however, begs the question: What is to be done with kinesthetic information about the hand when the target is presented only visually (V-VK) and what is done with visual information about the hand when the target is localized only kinesthetically (K-VK)? One possibility (not shown) is that the CNS simply ignores information about the hand in any sensory modality that is not also used to localize the target, relying only on sensory information that is directly comparable. Thus, only visual information about the hand would be used in the V-VK situation and only kinesthetic information about the hand would be used in the K-VK situation. But by doing so, one would forfeit the added precision that could be obtained by using both sources of sensory information about the hand holding the hammer. Alternatively, as illustrated in Figures 2E,F, the CNS could *reconstruct* the missing sensory information about the target by performing a cross-modal sensory transformation (green arrows). According to this arrangement, a kinesthetic representation of the target will be derived from visual information in V-VK, allowing both the visual and the kinesthetic information from the hand to be utilized. Analogously, the target can be reconstructed in visual space in K-VK, again allowing the comparison of target and hand to be carried out in both the visual and the kinesthetic domains.

The difference between the convergent and concurrent formulations becomes apparent if one compares the model predictions for V-VK versus K-VK in terms of the relative weighting given to visual or kinesthetic modalities. Consider first the concurrent models in Figures 2E,F. When computing the optimal weights λ_V_ and λ_K_ one must take into account not only the noise intrinsic to the sensory inputs, but also the noise added by cross-modal transformations (Soechting and Flanders, 1989; Tillery et al., 1991; Schlicht and Schrater, 2007) when a sensory input missing in one modality must be reconstructed from sensory signals in other. Taking into account this additional noise when applying Equation (6), one obtains for K-VK:

(7) $$\begin{array}{l} \lambda_{\text{V}}=\frac{\left(\sigma_{\text{T},\text{K}}^{2}+\sigma_{\text{H},\text{K}}^{2}\right)}{\left(\sigma_{\text{T},\text{K}}^{2}+\sigma_{\text{H},\text{K}}^{2}\right)+\left(\sigma_{\text{T},\text{K}}^{2}+\sigma_{\text{T},\text{K}\mapsto \text{V}}^{2}+\sigma_{\text{H},\text{V}}^{2}\right)}\\ \lambda_{\text{K}}=\frac{\left(\sigma_{\text{T},\text{K}}^{2}+\sigma_{\text{T},\text{K}\mapsto \text{V}}^{2}+\sigma_{\text{H},\text{V}}^{2}\right)}{\left(\sigma_{\text{T},\text{K}}^{2}+\sigma_{\text{H},\text{K}}^{2}\right)+\left(\sigma_{\text{T},\text{K}}^{2}+\sigma_{\text{T},\text{K}\mapsto \text{V}}^{2}+\sigma_{\text{H},\text{V}}^{2}\right)} \end{array}$$

and for V-VK:

(8) $$\begin{array}{l} \lambda_{\text{V}}=\frac{\left(\sigma_{\text{T},\text{V}}^{2}+\sigma_{\text{T},\text{V}\mapsto \text{K}}^{2}+\sigma_{\text{H},\text{K}}^{2}\right)}{\left(\sigma_{\text{T},\text{V}}^{2}+\sigma_{\text{T},\text{V}\mapsto \text{K}}^{2}+\sigma_{\text{H},\text{K}}^{2}\right)+\left(\sigma_{\text{T},\text{V}}^{2}+\sigma_{\text{H},\text{V}}^{2}\right)}\\ \lambda_{\text{K}}=\frac{\left(\sigma_{\text{T},\text{V}}^{2}+\sigma_{\text{H},\text{V}}^{2}\right)}{\left(\sigma_{\text{T},\text{V}}^{2}+\sigma_{\text{T},\text{V}\mapsto \text{K}}^{2}+\sigma_{\text{H},\text{K}}^{2}\right)+\left(\sigma_{\text{T},\text{V}}^{2}+\sigma_{\text{H},\text{V}}^{2}\right)} \end{array}$$

where σ^2^_T,K↦V_ and σ^2^_T,V↦K_ represent the noise added when reconstructing a visual representation of the target from kinesthetic information and the noise added when reconstructing the target in kinesthetic space from visual information, respectively. One can see from these sets of equations that changing what sensory information is available about the target has the potential of changing the weight given to each type of sensory feedback used to guide the hand. Indeed, less weight (smaller λ′s) will be given to the component comparisons that require the reconstruction of sensory information, due to the noise that these reconstructions add to the signals. In most cases, however, the weighting of the two component comparisons will shift toward the visual information when the target is visual (V-VK) and will shift toward the kinaesthetic domain when the target is kinaesthetic (K-VK). In the limit, if the transformation noise is very high compared to the input noise, the comparison that requires a sensorimotor reconstruction will be given zero weight, leaving only the direct comparison to drive the response.

For the convergent model, there is no inherent need to reconstruct sensory information that is not available. The CNS would simply use all the available sensory information about the target and all available sensory information about the hand in order to compute an optimal estimate of the position of each. This does not mean, however, that no sensorimotor transformations are required to implement the concurrent formulation. On the contrary, in order to combine spatial information from different sources, the different pieces of information must be expressed in a common reference frame R. Thus, for the convergent model, coordinate transformations will be required even though no “reconstruction” of missing sensory information is needed. These transformations will also add noise which will affect the weighting between the different inputs and should therefore be explicitly considered when comparing the concurrent and convergent models. According to Equations (1 and 5), the estimate of the hand's position and orientation will be based on a weighted sum of the visual and kinesthetic feedback, with the weight determined by the variance of the two feedback signals and by the noise added by the two sensorimotor transformations:

(9) $$\begin{array}{l} w_{\text{H},\text{V}}=\frac{\sigma_{\text{H},\text{K}}^{2}+\sigma_{\text{H},\text{K}\mapsto \text{R}}^{2}}{\sigma_{\text{H},\text{K}}^{2}+\sigma_{\text{H},\text{K}\mapsto \text{R}}^{2}+\sigma_{\text{H},\text{V}}^{2}+\sigma_{\text{H},\text{V}\mapsto \text{R}}^{2}}\\ w_{\text{H},\text{K}}=\frac{\sigma_{\text{H},\text{V}}^{2}+\sigma_{\text{H},\text{V}\mapsto \text{R}}^{2}}{\sigma_{\text{H},\text{K}}^{2}+\sigma_{\text{H},\text{K}\mapsto \text{R}}^{2}+\sigma_{\text{H},\text{V}}^{2}+\sigma_{\text{H},\text{V}\mapsto \text{R}}^{2}} \end{array}$$

One can see that even if one considers noise added by sensorimotor transformations, the convergent model, unlike the concurrent model, predicts that the weighting of sensory information will not change between V-VK and K-VK. Because the information available about the hand is the same in both V-VK and K-VK, the relative weight given to visual versus kinesthetic feedback about the hand will be the same in both circumstances, regardless of the sensory modality used to sense the target.

The convergent and concurrent models make two different predictions, therefore, about what happens when the modality of the target is changed while full feedback of the hand is available. These predictions allow one to differentiate between the two hypotheses experimentally. Indeed, a number of studies that have compared moving the hand to visual versus proprioceptive targets provide support for the hypothesis of concurrent comparisons shown Figure 1B. For instance:

- Sober and Sabes (2005) compared pointing to a visual target versus pointing with the one hand to the unseen index finger of the other. They used virtual reality to introduce conflict between visual and proprioceptive feedback about the initial position of the pointing finger. By measuring the bias toward the visual or the proprioceptive feedback about the position of the finger, they found a significant difference in the relative weighting of visual and kinesthetic hand feedback depending on the modality of the target.
- Sarlegna and Sainburg (2007) also used a virtual-reality technique to dissociate visual and proprioceptive feedback about the hand's initial position. The choice of target modality (moving to a visual target versus moving to the position of the other, unseen hand) had a significant effect on the contribution of vision versus proprioception to the control of the amplitude of rapid reaching movements.
- McGuire and Sabes (2009) made use of the well-known *retinal eccentricity effect* (Bock, 1986) and imposed changes in gaze direction to measure the reliance on visual versus kinesthetic information. They found that when visual and kinesthetic information about the hand was available, deviations due to changes in gaze direction, which would indicate coding of the movement in retinal space, depended on the target modality (pointing to a visual target versus pointing to the unseen left hand).
- Tagliabue and McIntyre (2011) asked subjects to align the hand with a target in the fronto-parallel plane. They used a virtual reality technique to introduce conflict between visual and kinesthetic reference frames during a memory delay. In these experiments the sensory modality used to present the target orientation had a significant effect on the weight given to visual versus kinesthetic comparisons when driving the response, with a shift toward visual information when the target was visual and kinesthetic information when the target was kinesthetic.

Because their data could not be reconciled with the encoding of movement parameters exclusively in either retinotopic space or kinesthetic space, the authors of the last two studies each proposed versions of the concurrent structure depicted in Figure 1B. The specifics of the models proposed by these different authors differ slightly from each other (more on the similarities and differences below) but both involve multiple comparisons in multiple reference frames and both can explain a shift in weighting toward visual information when the target was visible and toward kinesthetic information when the target was kinesthetic. Thus, compared to the hypothesis of convergent, multi-modal sensory integration shown in Figure 1A, the computational structure of multiple, concurrent comparisons depicted in Figure 1B provides a much more parsimonious explanation of the data reported from a number of different tasks and experimental paradigms.

## 3. To reconstruct or not to reconstruct?

Inherent to the concurrent model is the concept of sensory reconstruction. According to this idea, a visible target could be compared with proprioceptive information about the location of the hand if the visible information is transformed into proprioceptive space. Some such reconstruction would be necessary when, for instance, reaching toward a visual target with the unseen hand (V-K). The question remains, however, as to whether the visual target should be transformed into kinesthetic space or whether a visual representation of the hand should be constructed based on proprioceptive information from the arm. Transforming target information into kinesthetic space would be optional in a V-VK situation, where a direct comparison of target and hand could be carried out in visual coordinates. It would be even more superfluous to transform into visual space a purely kinesthetic (K-K) task. Yet the implication of visual representations in purely kinesthetic tasks is known to occur (Pouget et al., 2002b; Sober and Sabes, 2005; Sarlegna and Sainburg, 2007; McGuire and Sabes, 2009; Jones and Henriques, 2010). A key question to be addressed, therefore, is that of how the CNS chooses which comparisons to apply to a given task, and how to weight the different computations to arrive at the overall response. Under what conditions should information from one sensory modality be transformed into the reference frame of another?

In our original publication (Tagliabue and McIntyre, 2011) we argued that the CNS avoids sensory transformations, and thus performs direct comparisons whenever possible. Indeed, we observed that a V-VK task was carried out in visual coordinates while the equivalent K-VK task was carried out in kinesthetic space. (Note that we observed this result when subjects held their head upright. We saw a somewhat different result when subjects were asked to move their head during an imposed memory delay. We will discuss these latter results further down in this section). In our V-K and our K-V tasks, however, we observed that both visual and kinesthetic comparisons were performed, even though just one of these (and just one transformation) would have been sufficient. For instance, in V-K, subjects could have performed a single transformation of visual information into kinesthetic space, or they could have only transformed the kinesthetic hand information so as to perform the task in visual space. The fact that both transformations and both comparisons were performed shows that the CNS does sometimes perform “unnecessary” transformations beyond what would be minimally necessary to achieve the task.

In order to explain our results, and others, we had to resort to additional, albeit reasonable, assumptions that went beyond the basic tenets of MLE. The first was that direct comparisons are absolutely best, even though estimates of noise in the visual and kinesthetic channels and the conventional application of maximum likelihood would predict a more graded weighting between visual and kinesthetic information for the V-V and K-K tasks. The second was that the necessity of a single transformation would provoke the execution of a whole range of transformations into a number of different reference frame or sensory modalities. This could explain why the CNS would reconstruct a visual representation of a task that is otherwise purely kinesthetic, as was observed in the studies mentioned above. In the discussion of our results, we argued that this could be because a common neural network might generate the same amount of noise, whether performing one or many transformations. While this is a reasonable, and even testable, hypothesis, it still remains unproven and thus still constitutes, as of this writing, an *ad hoc* assumption that we had to invoke in order to reconcile empirical data with MLE.

In a more recent study, however, we showed how MLE *can* explain much, if not all, of the available data without these additional assumptions, if one properly accounts for co-variation of noise in sensory signals that have been reconstructed in one sensory modality from another (Tagliabue and McIntyre, 2013). The issue of co-variation is important because it conditions how two signals should be optimally weighted. If two signals are stochastically independent, the principle of maximal likelihood estimation says that the two quantities should be weighted according to the inverse of their respective expected variance. This weighted average will tend to reduce the effects of the independent noise in each component. But if the noise in one is correlated with the noise in the other, computing the weighted average will be less effective in reducing the overall noise. In the limit, if the noise in the two variables in perfectly correlated, then computing the weighted average will not reduce the overall noise at all.

To correctly compensate for covariance between two signals in the computation of the optimal weights to be applied, one must essentially take into account only the independent components of noise within each variable. In the case of two non-independent variables that exhibit Gaussian noise, the weighted combination of *x* and *y* that will minimize the variance of the output:

(10) z=λx+(1−λ)y

is given by the equation:

(11) $$\lambda =\frac{{\left(\sigma_{x}^{2}-{\text{cov}}_{x,y}\right)}^{-1}}{{\left(\sigma_{x}^{2}-{\text{cov}}_{x,y}\right)}^{-1}+{\left(\sigma_{y}^{-2}-{\text{cov}}_{x,y}\right)}^{-1}}$$

where cov_*x*,*y*_ is the covariance between x and y. Added insight can be achieved if one considers two components *x* and *y* are derived from two stochastically independent signals, *p* and *q* and a common component *c*:

(12) $$\begin{array}{l} x=p+c\\ y=q+c \end{array}$$

In this case, which is directly applicable to the sensorimotor transformations that are being considered in this paper, the covariance between *x* and *y* is precisely equal to the variance of the common component *c*:

(13) $$\begin{array}{l} \;\sigma_{x}^{2}=\sigma_{p}^{2}+\sigma_{c}^{2}\\ \;\sigma_{y}^{2}=\sigma_{q}^{2}+\sigma_{c}^{2}\\ {\text{cov}}_{x,y}=\sigma_{c}^{2} \end{array}$$

and Equation (11) reduces to:

(14) $$\lambda =\frac{\sigma_{q}^{2}}{\sigma_{p}^{2}+\sigma_{q}^{2}}$$

In other words, the optimal weighting of *x* and *y* depends only on the variance of the independent components *p* and *q*.

One can see from Equation (14) that if one of the two constituent signals presents only noise that is common to both quantities *x* and *y*, e.g.,:

(15) $$\begin{array}{l} x=p+c\\ y=c \end{array}$$

then the weight given to the constituent with the added noise (*x* in the example) will be zero. This fact can be used to predict when the CNS might reconstruct a representation of the task in a reference frame different from that of either the target localization or the feedback about the motor response. If the task allows for a direct comparison of target and effector information, e.g., when moving the hand to a remembered posture, the reconstructed comparison will contain all the variability of the kinesthetic inputs plus the noise added by the coordinate transformations while the direct comparison will contain no noise that is not also included in the reconstructed comparison:

(16) $$\begin{array}{l} \sigma_{\Delta \text{V}}^{2}=\sigma_{\text{T},\text{K}}^{2}+\sigma_{\text{H},\text{K}}^{2}+\sigma_{\text{T},\text{K}\mapsto \text{V}}^{2}+\sigma_{\text{H},\text{K}\mapsto \text{V}}^{2}\\ \sigma_{\Delta \text{K}}^{2}=\sigma_{\text{T},\text{K}}^{2}+\sigma_{\text{H},\text{K}}^{2} \end{array}$$

**Figure 3:**
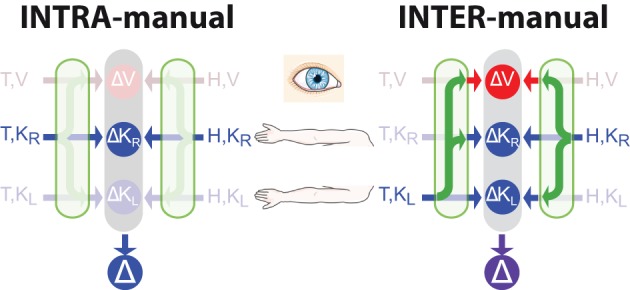
**Direct vs. indirect comparisons (modified from Tagliabue and McIntyre, 2013)**. The schematics represent the concurrent model applied to two tasks that are both purely kinesthetic (K-K). In the INTRA-manual task the subject feels the target position with the right hand (T,K_R_) and reproduces it with the same hand (H,K_R_). In the INTER-manual task the target is felt with the left hand (T,K_L_) and its position is reproduced with the right (H,K_R_). As in Figures 1, 2, red and blue arrows represent visual and kinesthetic signals, respectively, circular nodes represent movement vectors computed in different reference frames and green arrows represent sensory transformations. Each task can potentially be carried out partially in visual space by reconstructing a visual representation of the target (T,V) and a visual representation of the hand (H,V) from available kinesthetic inputs. In the INTRA-, but not INTER-manual task, a direct comparison between the kinesthetic signals about target and response is possible. Taking into account co-variance between reconstructed signals, only in the INTER-condition would a reconstruction of an “unnecessary” visual representation reduce movement variability. Grayed-out symbols represent sensory inputs that are absent in each task while grayed-out green arrows depict sensory reconstructions that are given no weight when MLE is applied.

Applying Equation (14) means that the comparison of the reconstructed signals, ΔV will be given no weight compared to the direct comparison ΔK. In other words, there is no advantage to transforming the task into an alternate reference frame (e.g., in visual space) in this situation. On the other hand, if the target and hand are sensed in two different reference frames, such that at least one sensory transformation is required, then reconstruction into a third reference frame might be beneficial. For example, if one is asked to reproduce with the right hand the remembered orientation of the left, a transformation will have to be applied to compare the hand orientation between the two limbs (see Figure 3), leading to the equations:

(17) $$\begin{array}{l} \;\sigma_{\Delta \text{V}}^{2}=\sigma_{\text{T},{\text{K}}_{\text{L}}}^{2}+\sigma_{\text{H},{\text{K}}_{\text{R}}}^{2}+\sigma_{\text{T},{\text{K}}_{\text{L}}\mapsto \text{V}}^{2}+\sigma_{\text{H},{\text{K}}_{\text{R}}\mapsto \text{V}}^{2}\\ \sigma_{\Delta {\text{K}}_{\text{L}}}^{2}=\sigma_{\text{T},{\text{K}}_{\text{L}}}^{2}+\sigma_{\text{H},{\text{K}}_{\text{R}}}^{2}+\sigma_{\text{H},{\text{K}}_{\text{R}}\mapsto {\text{K}}_{\text{L}}}^{2}\\ \sigma_{\Delta {\text{K}}_{\text{R}}}^{2}=\sigma_{\text{T},{\text{K}}_{\text{L}}}^{2}+\sigma_{\text{H},{\text{K}}_{\text{R}}}^{2}+\sigma_{\text{T},{\text{K}}_{\text{L}}\mapsto {\text{K}}_{\text{R}}}^{2} \end{array}$$

where K_L_ and K_R_ represent the kinesthetic information about the left and right hand, respectively. In this situation, each representation of the task, including representation that includes no direct inputs (ΔV) includes at least one source of noise that is independent from each of the others. Thus, one might expect to find that the task is carried out simultaneously in the intrinsic reference frame of each arm, and also in visual space. Indeed, when we compared precisely these two situations (matching the posture of the right hand to the remembered posture of the left versus matching the posture of the right hand to the remembered posture of the right hand) we observed exactly this behavior. The unilateral task showed no effect of deviations of the visual field, while the bilateral task did. This same reasoning can also be applied to a number of examples from the literature to explain why subjects appeared to reconstruct a visual representation of a task that could conceivably be carried out entirely in kinesthetic space (Pouget et al., 2002b; Sober and Sabes, 2005; Sarlegna and Sainburg, 2007; McGuire and Sabes, 2009; Jones and Henriques, 2010). Explicitly including the co-variation of reconstructed variable therefore increases the predictive value of the model structure depicted in Figure 1B.

## 4. The timing of sensory reconstructions

If one accepts the idea that the CNS transforms sensory information amongst multiple reference frames, one might also ask the question, when do such transformations occur? A number of studies have considered the performance of cross-modal transformations for the computation of a movement vector during planning (Sober and Sabes, 2003, 2005; Sarlegna and Sainburg, 2007; McGuire and Sabes, 2009; Burns and Blohm, 2010), but this is not the only time when such transformations may be needed. Sensory information about the target and limb continues to arrive throughout the movement, and the same issues about reference frames and sensor fusion arise when considering on-line corrections that are made based on this information. This question is of particular interest when one considers movements to memorized targets. In a V-K task, for instance, which is a task that requires at least one cross-modal sensory transformation, what happens if the target disappears before the reaching movement is started? How is the information about the target stored? Is it encoded in memory in visual space, to be transformed into kinesthetic space for comparison with proprioceptive information from the arm? Or is it immediately transformed into kinesthetic space and stored during the memory delay for later use?

**Figure 4:**
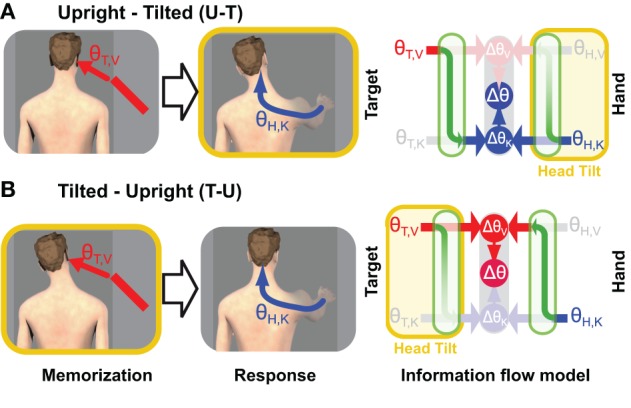
**Experimental manipulation of transformation noise (modified from Tagliabue et al., 2013)**. Two different experimental conditions are illustrated in which the subjects were asked to memorize the orientation (θ) of a visual target (red bar) and to reproduce it, after a delay, with their unseen hand. **(A)** In one condition (U-T) subjects memorized the target with the head upright and responded with the head tilted. **(B)** In the other condition (T-U), the target was memorized with the head tilted and the hand oriented with the head upright. On the right side of the figure are depicted the predictions of the Concurrent Model for each of the two experimental conditions. As in Figures 1, 2, and blue arrows represent visual and kinaesthetic signals, respectively and green arrows represent cross-modal transformations. Gray symbols represent sensory inputs that are absent. Because having the head tilted (yellow areas) causes cross-modal transformations to be significantly noisier, comparisons requiring such transformations are given less weight (faded green arrows) and comparisons for which sensory reconstructions are performed with the head upright are privileged.

The results of one of our recent experiments (Tagliabue et al., 2013) can be used to address this question. In that study we analyzed the V-K tasks alluded to above and illustrated in Figure 4. We asked subjects to perform this task in two different conditions, which differed only in terms of the timing of head movements. In one condition (U-T) subjects memorized the target with the head upright and produced the motor response with the head tilted. In the other condition (T-U) they memorized the target with the head tilted and moved the hand with the head upright. The rationale for performing this experiment with head tilted at different times is based on the notion that transformations between visual and kinesthetic space are disrupted (noisier) when the head is not aligned with gravity (Burns and Blohm, 2010; Tagliabue and McIntyre, 2011). This assumption is supported by a study of orientation matching between a visual and haptic stimuli (McIntyre and Lipshits, 2008). Whereas tilting the subject's entire body had no effect on visual-visual and haptic-haptic comparisons, responses were more variable in the case of a visual-haptic comparison when the body was tilted versus when it was upright. The fact that the inter-modal comparison became more variable, but not the intra-modal ones indicates that it is the transformation between sensory modalities, and not the actual sensory inputs, that are noisier when tilted with respect to gravity. In light of this fact, the relative weight given to visual information (λ_V_) in our more recent experiment and the overall variance (σ^2^_Δ_) will depend on whether each transformation is performed with the head upright or with the head tilted.

One can therefore differentiate between the different hypotheses Figure 4 as follows. For a V-K task we have:

(18) $$\begin{array}{l} \sigma_{\Delta \text{V}}^{2}=\sigma_{\text{T},\text{V}}^{2}\;+\sigma_{\text{H},\text{K}}^{2}+\sigma_{\text{H},\text{K}\mapsto \text{V}}^{2}\\ \sigma_{\Delta \text{K}}^{2}=\sigma_{\text{T},\text{V}}^{2}+\sigma_{\text{T},\text{V}\mapsto \text{K}}^{2}\;+\sigma_{\text{H},\text{K}}^{2} \end{array}$$

Taking into account the co-variation between a transformed signal and its source, as described in section 3, one can compute the weight given to the visual comparison:

(19) $$\lambda_{\text{V}}=\frac{\sigma_{\text{T},\text{V}\mapsto \text{K}}^{2}}{\sigma_{\text{T},\text{V}\mapsto \text{K}}^{2}+\sigma_{\text{H},\text{K}\mapsto \text{V}}^{2}}$$

and given the formula for the variance of a weighted sum of two variables that are not independent:

(20) $$\sigma_{ax+by}^{2}=a^{2}\sigma_{x}^{2}+b^{2}\sigma_{y}^{2}+2ab{\text{cov}}_{x,y}$$

the overall variance of the optimal estimate will be:

(21) $$\sigma_{\Delta }^{2}=\lambda_{\text{V}}^{2}\sigma_{\Delta \text{V}}^{2}+{\left(1-\text{​}\lambda_{\text{V}}\right)}^{2}\sigma_{\Delta \text{K}}^{2}+2\lambda_{\text{V}}\text{​}\left(1-\lambda_{\text{V}}\right){\text{cov}}_{\Delta \text{V},\Delta \text{K}}$$

(22) $$=\sigma_{\text{T},\text{V}}^{2}+\sigma_{\text{H},\text{K}}^{2}+\lambda_{\text{V}}^{2}\sigma_{\text{H},\text{K}\mapsto \text{V}}^{2}+{\left(1-\lambda_{\text{V}}\right)}^{2}\sigma_{\text{T},\text{V}\mapsto \text{K}}^{2}$$

(23) $$=\sigma_{\text{T},\text{V}}^{2}+\sigma_{\text{H},\text{K}}^{2}+\frac{\sigma_{\text{T},\text{V}\mapsto \text{K}}^{2}\sigma_{\text{H},\text{K}\mapsto \text{V}}^{2}}{\sigma_{\text{T},\text{V}\mapsto \text{K}}^{2}+\sigma_{\text{H},\text{K}\mapsto \text{V}}^{2}}$$

Now assume that the noise added when transforming from visual to kinesthetic or from kinesthetic to visual is the same, for a given orientation of the head, and that head tilt has the same additive effect on all transformations, i.e., we define:

(24) $$\sigma_{\text{T},\text{V}\mapsto \text{K}}^{2}=\sigma_{\text{H},\text{K}\mapsto \text{V}}^{2}=\sigma_{\mapsto }^{2}$$

when the transformation is performed with the head upright, and:

(25) $$\sigma_{\text{T},\text{V}\mapsto \text{K}}^{2}=\sigma_{\text{H},\text{K}\mapsto \text{V}}^{2}=\sigma_{\mapsto }^{2}+\sigma_{//}^{2}$$

when the transformation is performed with the head tilted to the side. Combining Equations (18–25), one can see that tilting the head will have no effect on λ_V_ if both transformations are performed with the head upright or both are performed with the head tilted:

(26) $$\begin{array}{l} \lambda_{\text{V}}{|}_{\text{up},\text{up}}=\frac{\sigma_{\mapsto }^{2}}{\sigma_{\mapsto }^{2}+\sigma_{\mapsto }^{2}}=\frac{1}{2}\\ \lambda_{\text{V}}{|}_{\text{tilt},\text{tilt}}=\frac{\sigma_{\mapsto }^{2}+\sigma_{//}^{2}}{\sigma_{\mapsto }^{2}+\sigma_{//}^{2}+\sigma_{\mapsto }^{2}+\sigma_{//}^{2}}=\frac{1}{2}\\ \lambda_{\text{V}}{|}_{\text{up},\text{up}}=\lambda_{\text{V}}{|}_{\text{tilt},\text{tilt}} \end{array}$$

Performing both transformations with the head upright or both with the head tilted will, however, have an effect on the overall variability:

(27) $$\begin{array}{l} \sigma_{\Delta }^{2}{|}_{\text{up},\text{up}}=\sigma_{\text{T},\text{V}}^{2}+\sigma_{\text{H},\text{K}}^{2}+\frac{\sigma_{\mapsto }^{2}\sigma_{\mapsto }^{2}}{\sigma_{\mapsto }^{2}+\sigma_{\mapsto }^{2}}\\ \;=\sigma_{\text{T},\text{V}}^{2}+\sigma_{\text{H},\text{K}}^{2}+\frac{\sigma_{\mapsto }^{2}}{2}\\ \sigma_{\Delta }^{2}{|}_{\text{tilt},\text{tilt}}=\sigma_{\text{T},\text{V}}^{2}+\sigma_{\text{H},\text{K}}^{2}+\frac{\left(\sigma_{\mapsto }^{2}+\sigma_{//}^{2}\right)\left(\sigma_{\mapsto }^{2}+\sigma_{//}^{2}\right)}{\sigma_{\mapsto }^{2}+\sigma_{//}^{2}+\sigma_{\mapsto }^{2}+\sigma_{//}^{2}}\\ \;=\sigma_{\text{T},\text{V}}^{2}+\sigma_{\text{H},\text{K}}^{2}+\frac{\sigma_{\mapsto }^{2}+\sigma_{//}^{2}}{2}\\ \sigma_{\Delta }^{2}{|}_{\text{tilt},\text{tilt}}=\sigma_{\Delta }^{2}{|}_{\text{up},\text{up}}+\frac{\sigma_{//}^{2}}{2}\\ \sigma_{\Delta }^{2}{|}_{\text{tilt},\text{tilt}}>\sigma_{\Delta }^{2}{|}_{\text{up},\text{up}} \end{array}$$

On the other hand, if one of the transformations is performed with the head upright, and the other with the head tilted, the opposite pattern should be observed. The weight given to visual information will depend on whether the transformation T,V ↦ K is performed with the head upright and the transformation H,K ↦ V is performed with the head tilted (up,tilt), or vice versa (tilt,up):

(28) $$\begin{array}{l} \lambda_{\text{V}}{|}_{\text{up},\text{tilt}}=\frac{\sigma_{\mapsto }^{2}}{\sigma_{\mapsto }^{2}+\sigma_{\mapsto }^{2}+\sigma_{//}^{2}}=0\text{as}\sigma_{//}^{2}\to \infty \\ \lambda_{\text{V}}{|}_{\text{tilt},\text{up}}=\frac{\sigma_{\mapsto }^{2}+\sigma_{//}^{2}}{\sigma_{\mapsto }^{2}+\sigma_{//}^{2}+\sigma_{\mapsto }^{2}}=1\text{ as }\sigma_{//}^{2}\to \infty \\ \lambda_{\text{V}}{|}_{\text{up},\text{tilt}}<\lambda_{\text{V}}{|}_{\text{tilt},\text{up}} \end{array}$$

while one would expect to see similar levels of overall variability between the two conditions, because in both cases one transformation is performed with the head tilted and one with the head upright:

(29) $$\begin{array}{l} \sigma_{\Delta }^{2}{|}_{\text{up},\text{tilt}}=\sigma_{\text{T},\text{V}}^{2}+\sigma_{\text{H},\text{K}}^{2}+\frac{\sigma_{\mapsto }^{2}\left(\sigma_{\mapsto }^{2}+\sigma_{//}^{2}\right)}{\sigma_{\mapsto }^{2}+\sigma_{\mapsto }^{2}+\sigma_{//}^{2}}\\ \sigma_{\Delta }^{2}{|}_{\text{tilt},\text{up}}=\sigma_{\text{T},\text{V}}^{2}+\sigma_{\text{H},\text{K}}^{2}+\frac{\left(\sigma_{\mapsto }^{2}+\sigma_{//}^{2}\right)\sigma_{\mapsto }^{2}}{\sigma_{\mapsto }^{2}+\sigma_{//}^{2}+\sigma_{\mapsto }^{2}}\\ \sigma_{\Delta }^{2}{|}_{\text{up},\text{tilt}}=\sigma_{\Delta }^{2}{|}_{\text{tilt},\text{up}} \end{array}$$

Note that the results remain valid even if σ^2^_T,V↦K_ ≠ σ^2^_H,K↦V_, for plausible values of σ^2^_T,V↦K_, σ^2^_H,K↦V_, σ^2^_T,V_, σ^2^_H,K_ and σ^2^_//_.

**Figure 5:**
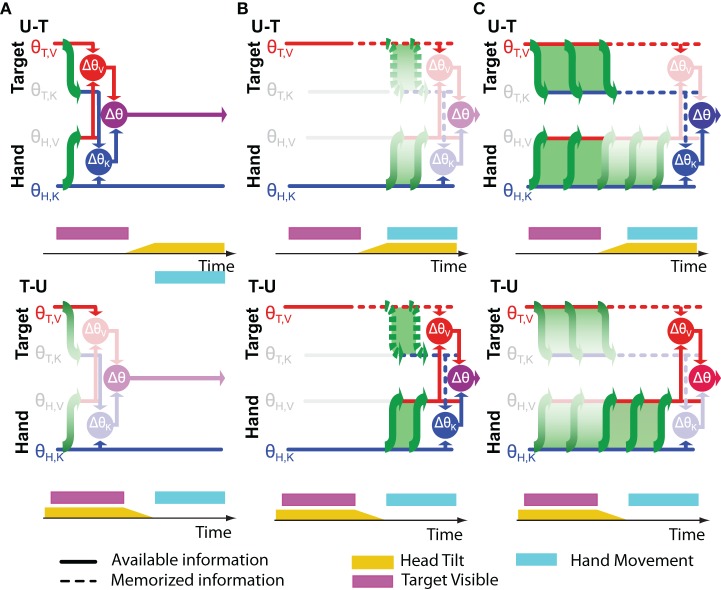
**Timing of cross-modal reconstructions**. Hypotheses concerning the time course of sensorimotor reconstructions are represented for the task depicted in Figure 4. The visibility of the target (purple bar) and the tilt of the head (yellow bar) are shown as time progresses from left to right. The hand moves only after the rotation of the head is terminated. Horizontal lines represent internal representations of the target (θ_T,V_ and θ_T,K_) and of the hand (θ_H,V_ and θ_H,K_). Gray symbols indicate sensory inputs that are absent, while green arrows indicate cross-modal reconstructions that may be performed. Vertical arrows and nodes indicate when the comparisons of target and hand are carried out, according to three hypotheses: **(A)** Cross-modal reconstructions and concurrent target-hand comparisons (Δθ_V_,Δθ_K_) are performed while the target is visible and the resulting movement vector (Δθ) is maintained and updated through the end of the movement. **(B)** Cross-modal reconstructions are performed during movement execution, relying on sensory inputs about the target stored in memory. **(C)** Cross-modal reconstructions are performed continuously as long as the sensory input is present; direct and reconstructed target representations are maintained in memory in parallel through the end of the movement. Faded nodes indicate target-hand comparisons that are noisier because they rely on cross-modal reconstructions that were performed with the head titled. Hypotheses **(A,B)** predict similar weighting of visual and kinesthetic information, and thus partial deviations of the response in both the U-T and T-U conditions, while hypothesis **(C)** predicts a significantly larger weighting of the visual comparison in the T-U than in the U-T conditions.

Using these mathematical considerations and the results of our experiment, one can distinguish between the three hypotheses about the timing of sensory reconstructions shown in Figure 5. If the movement vector is computed while the target is still visible (Figure 5A), then both transformations (T,V ↦ K and H,K ↦ V) will be performed with the head upright in the U-T condition and both will be performed with the head tilted in the T-U condition. According to Equations (26 and 27), the relative weight given to visual information should not change between the U-T and T-U conditions, while the overall variance should be greater for T-U than for U-T. Neither of these predictions is consistent with our empirical results in which we observed a significantly greater weight given to visual information in the T-U condition, compared to U-T, and similar levels of overall variability for both (Tagliabue et al., 2013). Note that this hypothesis can also be rejected by the strong effect of response modality that we observed in our previous study (Tagliabue and McIntyre, 2011). In all conditions tested in that study (K-K, K-VK, K-V, V-K, V-VK, and V-V) the subject's hand was outside the field of view during the time when the target was being presented. Therefore in all conditions the information available about the hand's orientation during target observation was *de facto* the same. If Figure 5A were correct, we would not have observed the strong effect of response modality on the weight given to visual versus kinesthetic information.

Figure 5B depicts an alternative hypothesis by which the CNS performs the requisite coordinate transformations starting at movement onset, relying on visual memory of the target after it disappears. In this case both transformations (T,V ↦ K and H,K ↦ V) would be performed with the head upright in the T-U condition and with the head tilted in the U-T condition. Applying once again Equations (26 and 27), one would expect to see similar weight given to visual information in both conditions and a significant difference in the overall variability, although according to this hypothesis, the higher variability would occur for U-T. As before, the empirical observations (Tagliabue et al., 2013) do not match the predictions of Figure 5B.

Our experimental findings can, however, be reconciled with a hypothesis by which cross-modal reconstructions of target and hand occur continuously, but *only long as the sensory input to be transformed is present* (Figure 5C). When the target disappears, as in our experiments, further reconstruction of its kinesthetic orientation from visual information is halted, and the remembered orientation is maintained in both spaces. Transformation of the continuously available hand kinesthesia into the visual domain proceeds, however, through the end of the movement. Here we fall into the situation in which the sensory transformations potentially used to control the movement do not all occur with the head at the same orientation. In the U-T condition, the last transformation of the target into kinesthetic space will occur with the head upright, while the latest transformations of the hand into visual space will occur throughout the movement, i.e., with the head tilted. Conversely, in the T-U condition, the last transformation of the target will occur with the head tilted, and the latest transformations of the hand with the head upright. Applying Equations (28 and 29), one expects to see a greater reliance on visual information in T-U than in U-T, with similar levels of overall variability between the two conditions, precisely as we observed (Tagliabue et al., 2013).

To summarize, we have shown that the reconstruction of sensory signals in alternate reference frames appears to occur only while the primary sensory input is available. An important corollary to this conclusion is that the CNS will also store spatial information concurrently in multiple reference frames, a prediction that can, in theory, be tested experimentally.

## 5. Generalized convergent and concurrent models

In the preceding sections we have discussed how the CNS might benefit from performing multiple, concurrent comparisons when, for instance, bringing the hand into alignment with a target. This discussion has highlighted a number of pertinent issues, including the evidence for single versus multiple comparisons, the importance of considering co-variation of signals when computing weights based on maximum likelihood and the timing of inter-modal transformations. The preceding sections leave open a number of questions, however, about when the various input signals are combined and about how to extend these concepts to situations where more than two sensory modalities may be involved. In this section we will formalize the distinction between convergent versus concurrent structures. In the section that follows we will show how the various computational concepts can be broadened to include questions such as how the CNS makes use of intrinsic versus extrinsic reference frames.

### 5.1. Fully convergent model

**Figure 6:**
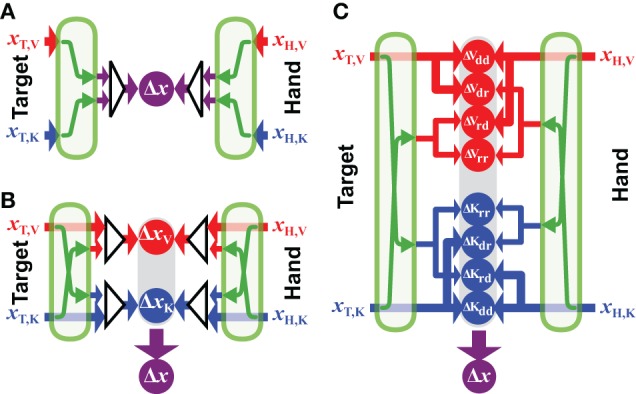
**Generalized models**. Three possible formulations of the sensorimotor integration model, all based on principles of maximum likelihood. **(A)** *Fully-convergent model*: first, the position of the target and hand are optimally estimated independently; then, these optimal estimations are compared in a unique reference frame to compute a single movement vector Δ*x*. Green arrows represent the sensory transformations necessary to encode the signals in a common reference frame before they can be combined. **(B)** *Hybrid-convergent/concurrent model*: available sources of information about the target are combined to build optimal estimations of its position in different reference frames. Ditto for the available hand information. Target-hand comparisons are then performed in each of these reference frames (Δ*x*_V_ and Δ*x*_K_) and the results of these comparisons are optimally combined to produce the net movement vector (Δ*x*). **(C)** *Fully-concurrent model*: available sources of information about target and hand are used to build concurrent target-hand comparisons in various reference frames. Information directly available in a given reference frame can be compared with both information directly available in the same reference frame and with information reconstructed from signal initially encoded in a different reference frame. It follows that, for each reference frame, all combinations between direct (*d*) and reconstructed (*r*) signals may be used to perform comparisons: ΔV_*dd*_, ΔV_*dr*_, etc.

Figure 6A shows the computational structure of the fully convergent model. A maximum likelihood estimate is made from all available inputs about the target's position and a similar process is applied to all available information about the position of the hand. As pointed out in section 2.1, the various sources of information must be transformed into a common reference frame in order for these optimal estimates to be computed and these transformations add noise. The calculations that describe the convergent model are therefore given by:

(30) $$\Delta x=\sum_{i=1}^{n}w_{\text{T},i}\Psi_{i\to r}\left(x_{\text{T},i}\right)-\sum_{j=1}^{m}w_{\text{H},j}\Psi_{j\to r}\left(x_{\text{H},j}\right)$$

where *x*_T,*i*_ and *x*_H,*j*_ are the sensory inputs about the target position in reference frame *i* and the hand position in reference frame *j*. Each input is associated with its own intrinsic variability (σ^2^_T,*i*_ or σ^2^_H,*j*_). The operator Ψ_*a*→*r*_ represents the a transformation of a position value from some reference frame *a* into the common reference frame *r*. Applying Ψ_*a*→*r*_ to an input value expressed in its intrinsic coordinate frame *a* creates a new value in the reference frame *r* with noise equal to the sum of the variance of the input (e.g., σ^2^_T,*a*_) and the variance added by the transformation (σ^2^_*a*→*r*_). Note that the common reference frame *r* could be some abstract reference frame that is independent from any given sensory frame, or it could be one of the *n* reference frames intrinsic to the sensory modalities used to sense the target position or one of the *m* reference frames used to sense the hand position. In this latter case, no transformation will be required for at least one sensory input, and we define Ψ_*r*→*r*_(*x*) = *x* and σ^2^_*r*→*r*_ = 0.

### 5.2. Hybrid convergent/concurrent model

According to the model presented in Figure 6B, it is presumed that the CNS will use all available information to represent the task in each of the component reference frames, and will then concurrently compare the target to the hand within each reference frame, before combining the results of each comparison to drive the motor response. We base this formulation on the model proposed by McGuire and Sabes (2009) for the combination of visual and kinesthetic information. From their discussion: *movements are always represented in multiple reference frames*, and from the Methods: *the model first builds internal representations of fingertip and target locations in both retinotopic and body-centered reference frames. These representations integrate all available sensory signals, requiring the transformation of non-native signals*. Extending these concepts to more than two sensory modalities and reference frames, the equation describing this formulation is:

(31) $$\Delta x=\sum_{i=1}^{N}\lambda_{i}\text{​}\left(\sum_{j=1}^{n}w_{i,\text{T},j}\Psi_{j\to i}\text{​}\left(x_{\text{T},j}\right)\text{​}-\text{​}\sum_{j=1}^{m}w_{i,\text{H},j}\Psi_{j\to i}\left(x_{\text{H},j}\right)\right)\;\;\;$$

where *N* is the total number of reference frames for which the comparison between target and hand will be made, *n* ⩽ *N* is the number of reference frames in which target information is directly available and *m* ⩽ *N* is the number of reference frames in which hand feedback is available. Implicit in this formulation is the idea that the CNS will always reconstruct sensory signals across modalities, even when sensory information is directly available within a given modality. One can see that this formulation allows for two sets of weights, those that determine the weight given to direct and reconstructed inputs within each reference frame [*w*_*i*,T,*j*_ and *w*_*i*,H,*j*_, comparable to the weights *w* described in the convergent model of Equation (1)] and those used to combine the results of the differences computed in each reference frame [comparable to the weights λ in the concurrent model of Equation (2)]. So, for instance, if both visual and kinesthetic information is available about the target, both the direct visual input and a transformed version of the kinesthetic information will be used to construct a representation of the target in visual space. Similarly, both the direct sensory input and the reconstructed visual input will be used to construct a representation of the target in kinesthetic space. The weight given to each source of information, however, will take into account the noise added by the cross-modal transformations. Thus, the representation of the movement in visual space will give more weight to the direct visual input than to the visual representation that is reconstructed from kinesthetic signals, etc. According to this model, the CNS will read out the desired movement vector by combining the differences computed concurrently in each reference frame, also according to the expected variance of each of the differences.

### 5.3. A fully-concurrent model

Here we propose a third formulation, shown in Figure 6C, based on the concept that individual comparisons form the building blocks for multisensory control of hand-eye coordination. According to this proposal, each available sensory input may be transformed into any and all other potential reference frames, as in the hybrid model described above. The two models differ, however, in terms of how the various reconstructions are handled within each reference frame. According to the fully concurrent model, the direct and reconstructed signals are not combined into a single representation of the target and of the hand within each reference frame. Rather, the CNS would compute individually the differences between all possible permutations of target and hand representations, both direct and reconstructed, within each reference frame, on a pair-by-pair basis. Only then would the results of all the individual differences be combined through a weighted average according to MLE in order to compute the movement vector. The computations that describe such a fully distributed, concurrent model, based on individual differences can be described by:

(32) $$\Delta x=\sum_{i=1}^{N}\sum_{j=1}^{n}\sum_{k=1}^{n}\gamma_{i,j,k}\left(\Psi_{j\to i}\left(x_{\text{T},j}\right)-\Psi_{k\to i}\left(x_{\text{H},\text{K}}\right)\right)$$

A simple mathematical convenience serves to adapt Equation (32) to situations where direct sensory inputs about the target or the hand are missing in one or more of the *n* sensory modalities. According to MLE, a given signal is weighted according to the inverse of its expected variance. If the quantity 1/σ^2^ is a measure of the confidence that one has in a given signal—i.e., the greater the variability, the lower the confidence—one can therefore assign to a missing sensory input an infinite variance, in the sense that the confidence in a missing signal will be 1/σ^2^ = 1/∞ = 0. By doing so, the weight given to a missing input, or to a transformed version of a missing input will automatically fall to zero in the calculations derived from MLE.

Note that Model 6C is “fully connected”, allowing for the possibility that, for instance, the CNS will reconstruct and compare kinesthetic signals in a visual reference frame even though both target and hand may be visible. This means that there may be multiple comparisons of the target and hand within any one reference frame due to the reconstruction from more than one other reference frames. Nevertheless, given the noise inherent to the reconstruction, the application of MLE will favor the comparison of the directly sensed visual signals within the each reference frame, when such direct information is available. Indeed, some components may drop out of the equation because MLE gives them a weight of zero, as we will see in the following.

## 6. Extrinsic reference frames

In the examples given above we have focused mainly on intrinsic reference frames native to the sensory modalities used to localize the target and the hand. This is due in part to the fact that the most widely documented studies of sensor fusion for eye-hand coordination, including those cited above, have considered two main reference frames: retinal for visual information and body centered for kinesthetic (a.k.a. proprioceptive) information. Depending on the task, however, other non-native reference frames are almost certainly of interest. For instance, ample evidence exists for the encoding of limb movements (Soechting and Ross, 1984; Darling and Gilchrist, 1991; Borghese et al., 1996; Luyat et al., 2001; Darling et al., 2008) or visual stimuli (Asch and Witkin, 1948b; Luyat and Gentaz, 2002) in a gravitational reference frames, as well as the encoding of information with respect to visual landmarks (Asch and Witkin, 1948a). In the following we examine the question of whether or not to make use of extrinsic reference frames in the context of each of the three models shown in Figure 6.

The convergent model of Figure 6A can accommodate the recoding of a sensorimotor task by realizing a change in the common reference frame *r*. Thus, the CNS may choose to combine sensory inputs in one possible reference frame or another, depending on the task conditions. Nothing in Equation (30), however, says anything about how *r* is chosen. Additional rules, not specified in Equation (30), would have to be found to resolve this outstanding question. As such, Model 6A is incomplete. Models 6B,C provide more elegant solutions to this question. An astute reader will have noticed the distinction between the lowercase *n* and *m* in Equations (31 and 32), representing the number of sensory inputs, from the uppercase *N* indicating the number of reference frames in which the comparison of target and hand is performed. These numbers could all be the same, but the two formulations allow for the use of additional reference frames not directly linked to a sensory input as well. According to these equations, each sensory input may be reconstructed in additional, non-native reference frames. Candidates include other, derived egocentric references such as the head or the shoulder or with respect to external references such as gravity or visual landmarks.

From the perspective of minimizing variability, however, recoding of sensory information in a non-native reference frame would not necessarily be advantageous, because the transformation of the information from a native to a non-native reference introduces additional noise. For instance, the variability of a visual target encoded with respect to gravity will include the variability of both retinal signals and of graviceptors. Moreover, all the variance of the target-hand comparison in the retinal reference frame will be included in the comparison encoded in the external reference frame. According to the analysis presented in section 3 the weight given to the external representation would drop to zero. One might therefore surmise that the recoding of spatial information in non-native reference frames will be avoided, when possible, in deference to direct comparisons of sensory information within the intrinsic reference frame of the different neural receptors. As we will show in the following examples, however, the native sensory representations may be affected by additional sources of noise, depending on the circumstances. The principle of maximum likelyhood coupled with the concurrent structures of Models 7B,C, can then predict which of the *N* reference frames, intrinsic or extrinsic, come into play in any given situation.

**Figure 7:**
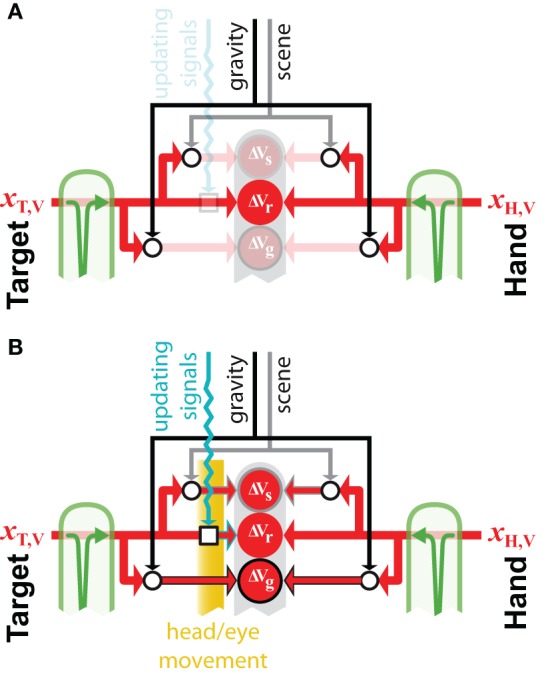
**External reference frames**. Example of how external sources of information, such as gravity and the visual scene, can be combined to build external encodings of initially retino-centric signal about the target, *x*_T,V_ and the hand, *x*_H,V_. Open circular and square nodes represent the recoding of information with respect to an external reference (circles) or the updating of egocentric information to account for movements of the body. All other symbols for inputs and transformations are as defined in previous figures. **(A)** If no movement occurs after the memorization of the retinal information about the target, its direct comparison with the retinal signal about the hand is possible, therefore encoding these signal with respect to the external gravitational and scene references would not reduce movement variability. **(B)** If the head moves in space, or if the eye moves within its orbit, a direct comparison between retinal signals about the target and hand is not possible, because the retinal information about the target must be updated to take into account the sensor movement. In this case, encoding the initially retino-centric signals with respect to the gravity and visual scene become advantageous, because the egocentric and the external encodings become partially uncorrelated.

### 6.1. External reference frames

Figure 7 shows an example of how the concurrent models may be applied to the question of whether or not to make use of an external reference frame for a given task. The model predicts that if the target and the hand can be sensed through the same modality and no movement of the sensor occurs between target memorization and response (Figure 7A), the brain should privilege a direct egocentric encoding of the movement. Since the transformation into the alternative reference frame would add noise, maximum likelihood will give the most weight to the direct comparison. This effect is amplified if one considers the co-variation between direct and reconstructed signals. Because a comparison performed in any other reconstructed reference frame would co-vary precisely with the inputs to the direct comparison, performing these additional encodings would not reduce the variability of the movement at all. On the other hand, if a movement occurs after the target is stored in memory (Figure 7B), an egocentric memory of the target would need to be updated to account for the sensor displacement (Droulez and Berthoz, 1992; Duhamel et al., 1992; Medendorp et al., 2008). In this situation, reconstructing additional, external encodings of the movement becomes advantageous, because the noise added by the updating of the intrinsic representation becomes comparable to the noise added when reconstructing in an external reference frame. This is especially true when the noise in the information used to update the egocentric representation of the target and the noise in the signals used as external references are independent.

The parallel structures of Models 6B,C are interesting because they provide a theoretical basis for using a combination of intrinsic and extrinsic reference frames, which appears to well correspond to behavioral (Burgess et al., 2004; Vidal et al., 2004; Burgess, 2006; Byrne et al., 2010) and physiological (Dean and Platt, 2006; Zaehle et al., 2007) evidence. Indeed, in a task of reaching with the outstretched hand for a visual or kinesthetic target, with visual or kinesthetic feedback about the response, or both, we were unable to reconcile empirical data with a computational model that relied on intrinsic reference frames alone (Tagliabue and McIntyre, 2012). We surmised that due to the movement of the head in our experiment, subjects encoded the task in external reference frames as well. Psychophysical studies have also shown that subjects tend to use egocentric representations if they remain stable after memorization, but they combine egocentric and external representations if their body moves (Burgess et al., 2004; Burgess, 2006). Similarly, during reaching to visual targets, external visual landmarks appear to be neglected if the hand visual feedback is reliable; whilst they are integrated to build an allocentric representation of the movement if the hand visual feedback was absent or unpredictable (Obhi and Goodale, 2005; Neely et al., 2008).

### 6.2. Memory

The need to store target information in memory for some time before the movement occurs can also motivate the transformation of sensory information into a non-native reference frame. In eye-hand tasks with imposed memory delays, the variability of responses tends to increase with the length of the delay (McIntyre et al., 1997, 1998). Thus, the simple act of storing spatial information in memory adds noise. According to the hypothesis related in section 4, the target location will be stored in memory simultaneously in more than one reference frame. Assuming that each representation of the remembered target position will degrade independently (i.e., each will accumulate noise that is stochastically independent from the other), it becomes more and more interesting, a maximum likelihood perspective, to make use of the non-native representations, despite the added cost of reconstructing those representations in the first place. This reasoning is supported by a study in which subjects were asked to point to targets located along a straight line in 3D space (Carrozzo et al., 2002). As the memory delay increased, patterns of variability of the pointing position were more-and-more constrained by the extrinsic reference provided by the direction of the line in 3D space. This can be interpreted as a shift in weighting between egocentric and allocentric reference frames, even when the body does not move. By simply substituting “memory processes” for “head/eye movement”, however, Figure 7 can be used to understand why the CNS may rely more on the encoding of a task in a external reference frame when memory processes are involved.

## 7. Discussion

In this paper we have described three analytical models (see Figure 6) that share a number of defining features. One of these, the idea that the CNS can express spatial information in multiple reference frames while transforming information between them, is a common theme that is supported by numerous theoretical and experimental studies. To cite a few examples, Droulez and Cornilleau-Peres (1993) proposed a distributed model of “coherence constraint” by which spatial information may be encoded in reference frames intrinsic to each sensor and they described a computational structure by which information from one sensor can be reconstructed based on redundant information from other sensors when the primary source is not available. Bock (1986) identified a phenomenon of bias when pointing to targets that lie at a location peripheral to the center of gaze. This phenomenon has been used in a number of studies to argue that whether pointing to visual, auditory or even proprioceptive targets, the CNS carries out the task in retinotopic coordinates (Enright, 1995; Henriques et al., 1998; Pouget et al., 2002b). These observations can be linked to neural properties through models that solve the problem of recoding information in different reference frames by using basis functions and attractor dynamics (Pouget et al., 2002a) or restricted Boltzmann machines (Makin et al., 2013).

The premise that the CNS combines sensory information based on relative variance has also found considerable experimental support: van Beers et al. (1996) showed that the precision of pointing movements increased when the subject could use both visual and kinesthetic feedback signals, compared to when only one sensory feedback modality was available. They also showed that the relative weight given to the two sensory signals depended on their relative variability (van Beers et al., 1999). Ernst and Banks (2002) varied experimentally the noise in the sensory signals available to subjects when they grasped a virtual object that provided both visual and haptic cues about size. Using verbal judgments, they showed how the overall perceptual response shifted toward the haptic information when the precision of the visual inputs was degraded. Smeets et al. (2006) assumed that the CNS maintains both a visual and a kinesthetic representation of targeted movements. When vision of the hand was allowed, this sensory modality dominated due to its higher precision. But when vision of the hand was occluded and subjects were asked to make consecutive movements, the authors observed a gradual shift toward a reliance on proprioceptive information, as indicated by gradual drift in the direction of biases that are specifically associated with this modality. They attributed this shift to a re-weighting toward proprioceptive information as the visual representation of the occluded hand degrades over the course of sequential movements.

These themes of transformations and maximum likelihood come together when one considers the noise added when converting sensory information from one reference frame to another. As alluded to in section 2.1, the added noise inherent to sensory information that is reconstructed from other sources will cause a shift toward the alternative, directly sensed information. This principle has given rise to other empirical manifestations: Sober and Sabes (2003, 2005) postulated that the CNS combines visual and proprioceptive information at two different stages in the planning of targeted hand movements. First, the movement vector is calculated in visual space as the difference between the position of the visual target and the initial position of the hand. Kinesthetic information about the hand's position is also used at this stage, but because it must be transformed into visual space, it is given much less weight, in accord with MLE. At a second stage, the visual movement vector is converted into a motor vector, based primarily on proprioceptive information, but also accommodating a weaker influence of visual information about the target, hand and limb configuration transformed into motor coordinates. Burns and Blohm (2010), using the same model structure as Sober and Sabes, observed a reduction of the weight given to proprioceptive information in the calculation of the movement vector during planning when the head was tilted in a V-VK task. They attributed the shift to the fact that (a) the movement vector was calculated in visual space, requiring that the proprioceptive information about hand position be transformed in order to be useful and (b) tilting the head with respect to gravity increases the noise added by manual-to-visual transformations, thus further decreasing the weight given to the reconstructed signals. Tagliabue et al. (2013) examined the effects of head tilt on the weighting of sensory information. In a V-K task (Figure 4), if the head was tilted during target acquisition, but not the motor response, the CNS gave greater weight to the visual representation, presumably because transforming the visual target into kinesthetic space with the head tilted would be much noisier than transforming kinesthetic information about the hand into visual space with the head upright. Conversely, if the head was held upright when the target was acquired, but the head was tilted during the motor response, then the task was carried out in kinesthetic space so as to avoid the kinesthetic-to-visual transformation that would have to occur while the head was tilted.

Although the three computational models of Figure 6 share a number of features, as described above, they vary in terms of the level of convergence or parallelism in the processing of sensory information. Model 6A presents the highest level of convergence, combining all available inputs about the target and all available inputs about the hand before calculating a movement vector based on the two optimal estimates. Model 6A provides no clue, however, as to what is the common reference frame for any given task, nor how the common reference frame might change from one task to another. Models 6B,C provide more elegant solutions to this question by allowing the comparison of target and hand to be carried out simultaneously in multiple reference frames. The same rules that determine which sensory inputs will dominate in any given situation (maximization of likelihood) also determine the weight given to the comparison carried out in each of the component reference frames. The computational scheme depicted in Figure 6B combines features of both the convergent model of Figure 1A and the concurrent model of Figure 1B. Whereas multiple comparisons of target and hand are performed in different reference frames, one can see nevertheless that there is a convergence of multimodal sensory signals about the target and about the hand before these two quantities are compared (subtracted) within each reference frame. In contrast, Model 6C combines the results of binomial comparisons of a single sensory input about the target (direct or reconstructed in another reference frame) with a single sensory input about the hand (also direct or reconstructed). Model 6C is the least convergent of the three and as such, lends itself to a modular approach to sensory integration for the coordination of eye and hand.

### 7.1. Model predictions

Which of the three models depicted in Figure 6 best represents human sensorimotor behavior and the underlying neurophysiology? The three computational structures that we have compared here can be distinguished on theoretical grounds and the differences between them lead to testable hypotheses, both at the behavioral level and in terms of the neural implementation as measured by electrophysiological or other methods.

#### 7.1.1. Fully convergent vs. concurrent

The question as to whether sensory signals are combined in a unique reference frame that is defined *a priori* (i.e., in line with Figure 6A) prior to performing the comparison between hand and target has received considerable attention in recent years and can, perhaps, already be rejected. From a Bayesian perspective, it can be argued that it is advantageous to maintain multiple representations of movement parameters, expressed in diverse reference frames, in order to optimize motor performance. Electrophysiological evidence also supports the notion that motor planning and execution is carried out in multiple reference frames in parallel, both across different regions of the brain and within a single cortical area (Buneo et al., 2002; Beurze et al., 2010; Buchholz et al., 2013; Maule et al., 2013; Reichenbach et al., 2014). At the behavioral level, the fully convergent model depicted in Figure 6A cannot predict certain experimentally observed characteristics of movement planning and execution. As explained in the earliest sections of this article (2–2.1), such a computational model cannot explain why sensory information about the hand is weighted differently between K-VK and V-VK tasks, nor would Model 6A be able to predict why the CNS would reconstruct a visual representation of kinesthetic pointing task when the task is bilateral, but not when it is unilateral (Tagliabue and McIntyre, 2013). Moreover, the combination of parallel comparisons in a variety of coordinate systems gives meaning to the concept of a *hybrid* reference frame (Carrozzo and Lacquaniti, 1994). Rather than considering that the task is executed in some abstract reference frame that has little or no physical meaning, one can instead understand that the characteristics of a so-called hybrid reference frame may in fact be the manifestation of a parallel, weighted combination of individual target-hand comparisons carried out in reference frames tied to identifiable objects or sensors.

Studies that have explicitly considered sensor fusion in the case of reaching or pointing tasks have often assumed, implicitly or explicitly, the fully convergent computational structure depicted in Figure 1A. One such example is the work carried out by van Beers et al. (1996, 1999) who postulated that a minimization of motor variability could be the driving factor behind the choice of one motor plan over another. They explicitly refer to a convergent maximum likelihood model structure along the lines of Equation (1). The work by Smeets et al. (2006) included the assumption that the CNS maintains both a visual and a proprioceptive representation of the hand and of the target, but did not include any explicit consideration of the transformation of visual information into proprioceptive space or vice versa. Furthermore, the equations that the authors used to make the model predictions in that study would appear to adhere to the computational structure evoked by the convergent model described by Equation (1). Nevertheless, the structure of concurrent comparisons described by Equation (2) can also accommodate both of these studies, without contradiction. Thus, even though Equation (1) has been used on occasion to explain the results of a number of studies, the ability of Equation (2) to explain those studies, and to also explain the effects of target modality that cannot be explained by Equation (1) means that Equation (2) provides a more parsimonious explanation of human sensorimotor behavior.

#### 7.1.2. Hybrid concurrent/convergent vs. fully concurrent

Experiments testing the two concurrent hypotheses (Figures 6B,C) have been performed by various groups and reported in the literature. We believe that the hybrid formulation of Equation (31) is representative of the model proposed by McGuire and Sabes (2009). These authors used a more sophisticated Bayesian analysis to formulate their hypothesis, but as they point out, the convolutions required to represent a coordinate transformation in Bayesian notation are simply additions or subtractions and if there is no prior to be taken into account, the posterior is proportional to the likelihood. This model has been used to interpret a number of empirical results (McGuire and Sabes, 2009, 2011; Burns and Blohm, 2010). In our own studies and publications, we have implicitly used the computational structure of Equation (32) to interpret the results of a series of experiments on multi sensory integration (Tagliabue and McIntyre, 2008, 2011, 2012, 2013; Tagliabue et al., 2013). But whereas both models have been used with success to explain a wide range of empirical results, the differentiation between the hybrid concurrent/convergent formulation of Figure 6B and the fully concurrent formulation in Figure 6C has not, to our knowledge, been explicitly taken up in the literature. Yet it should be possible to distinguish between the two mechanisms, both in terms of potential theoretical advantages of one computational scheme over the other and in terms of empirical results, as we will discuss below.

One key difference between Figures 6B,C is that of when the difference between target and hand is actually computed. In a linear system, this distinction is not very important, since Model 6B can be rearranged algebraically to match Model 6C, and vice versa. But evidence suggests that the combination of sensory signals occurs in a non-linear fashion, in part as a means to deal with sensory signals that may or may not come from the same stimulus or event (Roach et al., 2006; Knill, 2007; Hospedales and Vijayakumar, 2009). If sensory signals are separated in distance or in time, the Bayesian optimal may be to rely fully on one signal or the other, rather than an weighted sum of the two. A corollary of these non-linear processes is that as two redundant signal become more separated, the combined estimate may become noisier (Wallace et al., 2004). Model 6C has an advantage over 6B in this respect. By combining sensory signals only after computing the movement vector, disparity between reference frames will drop out, provided that the disparity is the same for the target and for the hand. One might therefore test this hypothesis by artificially modulating the disparity between reference frames. The prediction of Model 6C is that such an operation will not affect motor precision.

The question of how the CNS takes into account covariance between signals could also provide the basis for favoring one model over the other. In Model 6B, the combination of visual and kinesthetic information about the target are combined by using a “local” optimality criterion, that is by taking into account the variability of the signal to be combined (including the necessary cross-modal transformations), but neglecting how the resulting optimal estimation will be used in later stages. In particular, this local optimal weighting of the target information neglects the consequences of any covariance that may be generated between the two concurrent comparisons ΔV and ΔK. The very same considerations are valid, of course, for the hand information. It follows that the brain could tend to over-estimate the benefit of weighting a given signal, because, although it would “locally” provide a more precise estimation of the target and of the hand positions, “globally” it would increase the covariance between ΔV and ΔK, and if not corrected, will increase the variance of the final output. In other words, generating optimal estimates of target and hand does not necessarily lead to optimal targeted hand movements. Model 6C, on the other hand, is based on the combination of pairwise comparisons of target and hand, with maximum likelihood being applied to minimize the variability of the combination of multiple movement vectors. Through this more modular approach, it is potentially easier to identify and adjust for co-variation between movement vectors.

**Figure 8:**
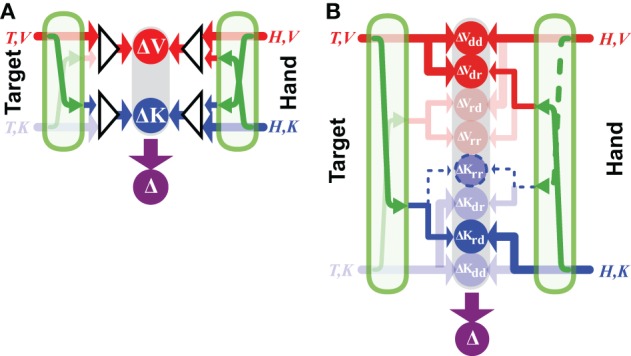
**Hybrid convergent/concurrent versus full concurrent**. Information flow predicted by the hybrid convergent/concurrent **(A)** and fully-concurrent **(B)** models for a V-VK condition in which the target can be sensed only visually, but the subject has both visual and kinesthetic information about the hand. Missing sources of information are represented by faded colors. Dashed lines represent sensory transformations and comparisons that can be neglected without a decrease in motor performance, given the extent to which the noise in these calculations correlates with the other comparisons. The fully-concurrent model, but not the Hybrid model, predicts that in the V-VK condition the reconstruction of the kinesthetic representation of the hand from visual feedback can be avoided.

An example of this is shown in Figure 8, in the case of a V-VK task. The hybrid model predicts that both visual and kinesthetic information about the hand will be used to construct representations of the hand in each of the two reference frames (Figure 8A). Due to the inter-modal transformations, the comparison carried out in kinesthetic space will be correlated with the comparison carried out in visual space. The optimal combination of ΔV and ΔK will need to be modified to take into account the resulting co-variation. Model 6C applied in this situation instead predicts that comparison of the visual target position, reconstructed in kinesthetic space, with the representation of the hand, reconstructed from visual information, will simply drop out, due to the co-variance with the direct comparison of target and hand in visual space (Figure 8B). One might therefore ask the question, will the CNS, like Penelope waiting for Ulysses with her weaving (Homer, VIII century BC), perform cross-modal reconstructions, only to undo their effects at a later stage (Figure 8A)? Or, by maintaining a more modular approach, can the CNS more efficiently achieve the optimal solution by performing only those transformations and comparisons that are beneficial in any given situation (Figure 8B)?

Of course the ultimate test of the hypotheses presented here would be to find correlates of models 6B or 6C in electrophysiological studies of neuronal activity. Model 6B predicts that one should find neurons that respond to multiple sensory inputs about the target and similar neurons encoding information about the hand. Model 6C makes a novel prediction that certain cells will be sensitive to inputs about the target in one (and only one) sensory modality but that the spatial information will be expressed in the coordinate frame of another. For example, Model 6C predicts the existence of a cell that encodes the movement vector in visual space, even though the cell may be sensitive to modulation of proprioceptive, but not visual, signals. This would not be the case for Model 6B, where sensory signals from each available sensory modality are expected to converge prior to the computation of the movement vector.

## 8. Conclusions

In this article we have formulated computational models that rely on multiple concurrent computations carried out in multiple reference frames in order to optimally drive the hand to a target. We have compared these concurrent models to the more conventional viewpoint that presupposes the use of a single, common reference frame for combining multi-sensory information. The concurrent models are attractive because of their modular structure and because they better explain a variety of empirical studies. Moreover, they place the question of how to combine sensory information and how to choose the reference frame(s) for any given task into a common theoretical framework, that of maximum likelihood estimation. They also make specific, testable predictions about the sensory transformations that are performed and the representations of target and hand that are maintained in working memory during the performance of sensorimotor tasks. In the spirit of this special issue on modularity in motor control, we therefore propose that the CNS performs multisensory integration in a highly modular fashion, building up the required motor commands for targeted movements from a principled combination of elementary target-hand comparisons.

### Conflict of interest statement

The authors declare that the research was conducted in the absence of any commercial or financial relationships that could be construed as a potential conflict of interest.
